# Meningitis and Bacteremia by Unusual Serotype of *Salmonella enterica* Strain: A Whole Genome Analysis

**DOI:** 10.1155/2024/3554734

**Published:** 2024-03-22

**Authors:** Thamer Brek, Gassem A. Gohal, Muhammad Yasir, Esam I. Azhar, Ibrahim A. Al-Zahrani

**Affiliations:** ^1^Medical Laboratory Sciences Department, Faculty of Applied Medical Sciences, King Abdulaziz University, Jeddah, Saudi Arabia; ^2^Public Health Laboratory, The Regional Laboratory and the Central Blood Bank, Jazan Health Directorate, Jazan, Saudi Arabia; ^3^Department of Pediatrics, Faculty of Medicine, Jazan University, Jazan, Saudi Arabia; ^4^Special Infectious Agents Unit-Biosafety Level-3, King Fahd Medical Research Centre, King Abdulaziz University, Jeddah, Saudi Arabia

## Abstract

**Background:**

Although meningitis caused by *Salmonella* species is relatively rare and accounts for <1% of the confirmed cases in neonates, it is associated with case complications and fatality rates up to 50–70% when compared to other forms of Gram-negative bacilli meningitis.

**Objectives:**

We conducted an investigation into the first reported case of neonatal meningitis caused by nontyphoidal *S. enterica* in Jazan, a region in the southwestern part of Saudi Arabia.

**Methods:**

CSF and blood culture were collected from a female neonate patient to confirm the presence of bacterial meningitis. WGS was conducted to find out the comprehensive genomic characterization of *S. enterica* isolate.

**Results:**

A 3-week-old infant was admitted to a local hospital with fever, poor feeding, and hypoactivity. She was diagnosed with Salmonella meningitis and bacteremia caused by *S. enterica*, which was sensitive to all antimicrobials tested. WGS revealed the specific strain to be *S. enterica* serotype Johannesburg JZ01, belonging to ST515 and cgMLST 304742.

**Conclusions:**

We presented a genomic report of rare case of NTS meningitis in an infant who is living in a rural town in Jazan region, Saudi Arabia. Further research is required to understand the impact of host genetic factors on invasive nontyphoidal Salmonella infection.

## 1. Introduction

*Salmonella* is an enteric, motile, and nonlactose fermenting Enterobacteriaceae. This facultative anaerobic organism can infect multiple animal hosts including humans via a wide variety of contaminated foods [1]. Human infections caused by *Salmonella enterica* cause serious disease burdens across the world. This widely distributed species is divided into more than 2500 distinct serovars based on O and H antigens, which are classified as typhoidal or nontyphoidal Salmonella (NTS) [2]. Typhoidal *Salmonella* are a group of *S. enterica* serotypes that cause typhoid (enteric) fever, such as typhi, paratyphi A, paratyphi B, and paratyphi C. While nontyphoidal *Salmonella* (NTS) refers to other *S. enterica* serotypes, particularly Enteritidis and Typhimurium which are considered the two most common serotypes reported in the United States [3]. Globally, it is commonly recognized that NTS serotypes are the leading cause of gastroenteritis, a localized infection of the terminal ileum and colon, and they are usually characterized by diarrhea and vomiting [4]. In addition to digestive disease, NTS infections can also invade normally sterile sites, resulting in bacteremia, meningitis, and other focal infections [5]. Moreover, invasive nontyphoidal *Salmonella* (iNTS) infection can be seen in patients with comorbidities, sickle cell anemia, and HIV infection, in infants and the elderly. In 2017, more than 530,000 cases of NTS invasive disease were reported, with the highest incidence in sub-Saharan Africa particularly in children younger than 5 years old [6].

Bacterial meningitis is an inflammatory disease of the central nervous system (CNS) which can be diagnosed by the presence of bacteria in the CSF. Despite advances in treatment, meningitis is still a disease of global dimension, which can end fatally or leave long-term neurological sequelae in survivors [7]. Furthermore, neonatal bacterial meningitis (NBM) is a devastating disease of newborns that can occur during the first four weeks of life. Major pathogens associated with neonatal meningitis include group B Streptococcus (GBS), *Escherichia coli*, *Listeria monocytogenes,* and other Gram-negative bacilli [8]. Reported mortality rates that resulted by these pathogens vary from 10% to 50%, based on world region, gestational age, and weight of the neonate [8]. Moreover, survivors of NBM often suffer severe neurological sequelae that may include seizures, hearing loss, cerebral palsy, and delayed development, occurring in 12% to 44% of survivors [9]. Although meningitis caused by *Salmonella* species is relatively rare and accounts for <1% of the confirmed cases in neonates, it is associated with case complications and fatality rates up to 50–70% when compared to other forms of Gram-negative bacilli meningitis [10].

The brain is well protected against microbial invasion by cellular barriers, such as the blood-brain barrier (BBB) and the blood cerebrospinal fluid barrier (BCSFB). In addition, cells within the central nervous system (CNS) are capable of producing an immune response against invading pathogens [7]. The penetration of these barriers by meningitis-causing bacteria is defined by a complex interaction between host cells and the bacterial pathogens, which use an array of virulence factors and inhibitors of immune response to facilitate invasion, intracellular survival, and systemic dissemination [7]. Here, we investigated the first report of nontyphoidal *S. enterica* causing neonatal meningitis in the southwestern region of Saudi Arabia. Whole genome sequencing (WGS) was conducted to find out the comprehensive genomic characterization of our isolate.

## 2. Methods

### 2.1. Identification and Antimicrobial Susceptibility Testing

As part of routine testing conducted in the microbiology laboratory of a tertiary hospital in Jazan, southwestern region of Saudi Arabia, CSF and blood culture were collected to confirm the presence of bacterial meningitis in female neonate patients. The primary bacterial growth in the blood culture was conducted using an automated blood culture system (BACT/ALERT 3D 120 combo, BioMerieux, USA). Identification and antibiotic susceptibility testing of the bacterial isolate from CSF and blood subculture were performed with the MicroScan WalkAway plus system (Beckman Coulter, USA). The results of antimicrobial susceptibility testing (AST) were interpreted according to the Clinical and Laboratory Standards Institute (CLSI) guidelines [11]. From pure culture on the MacConkey agar plate, the identified bacterial isolate was transferred into a 1.5 ml Eppendorf tube containing Luria–Bertani (LB) broth (HiMedia Labs, India) with 20% (v/v) glycerol and was maintained at −80°C for genomic analysis.

### 2.2. DNA Extraction

The bacterial strain was aseptically grown in Luria–Bertani (LB) broth overnight at 37°C. Genomic DNA from harvested culture was extracted using GeneJET Genomic DNA Purification Kit (Thermo Scientific, lithuania) according to the manufacturer's instructions. The yielded DNA sample was quantified using a NanodropTM 1000 spectrophotometer (Thermo Scientific, MA, USA) and stored at −20°C.

### 2.3. Genomic Sequencing and Data Analysis

**Figure 1:**
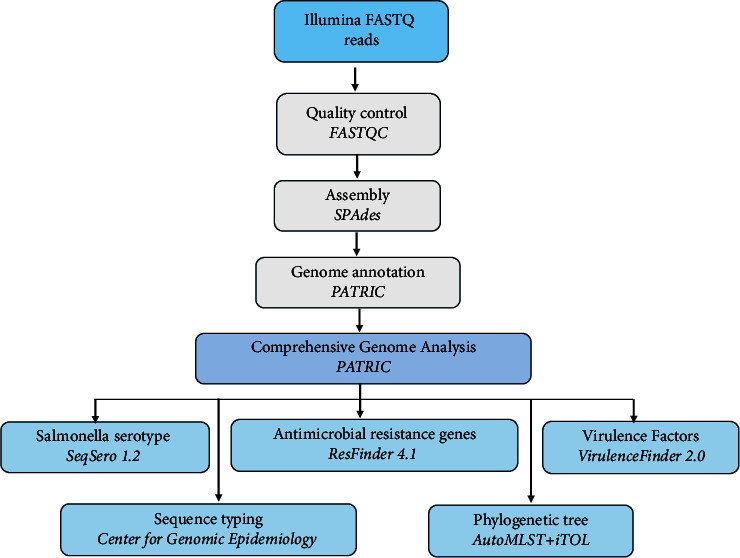
The bioinformatics workflow *for WGS analysis* is represented by boxes, each of which corresponds to a component that performs a series of functions. The major bioinformatics services used in each part are also mentioned (revealed in *italics*).

Whole genome sequencing of the *S. enterica* isolate was carried out as previously described by Yasir et al. [12] using a MiSeq system (Illumina Inc., San Diego, CA, USA) with v.1, 2 × 150-bp chemistry. Genome assembly was prepared from high-quality filtered reads using the SPAdes 3.9 algorithm. Genome annotation was carried out using the PATRIC web resources (https://www.patricbrc.org/). The *Salmonella* serotype was identified using SeqSero 1.2 through the Center for Genomic Epidemiology web server (https://cge.food.dtu.dk/services/SeqSero/). Antimicrobial resistance genes and virulence genes were identified using ResFinder 4.1 and VirulenceFinder 2.0, respectively. The sequence type (ST) and core genome MLST (cgMLST) of the isolate were identified using the Center for Genomic Epidemiology web server (https://cge.food.dtu.dk/services/MLST/). A phylogenetic tree was built with other closely related genomes of *S. enterica* obtained from NCBI based on multilocus sequence analysis (MLSA) using the AutoMLST web tool [13] and Species Tree v2.1.10 program, and the interactive Tree of Life tool (iTOL) was used to visualize the phylogenetic tree.  Figure 1 illustrates a bioinformatics workflow for WGS analysis.

## 3. Results

### 3.1. Case Summary

In August 2020, a 3-week-old female infant, who is living with her family in a rural town in the Jazan region, arrived at the emergency department of a local hospital with a clinical history of fever, poor feeding, hypoactivity with no vomiting, or diarrhea. She was born, as part of a twin, by vaginal delivery at full term of gestation, without any abnormalities being observed during pregnancy. On examination in the ER, her body temperature was 38.1°C, heart pulse rate 180 beats per minute, and blood pressure 75/40 mmHg. The primary laboratory examinations indicated a normal leucocyte count (6,410/*μ*L), a normal hemoglobin level (16.4 g/dL), and elevated serum C‐reactive protein (24 mg/L). Then, a nasopharyngeal swab, cerebrospinal fluid (CSF), and blood culture were requested for further clinical investigation. Intravascular injections of ampicillin, cefotaxime, and paracetamol were given as primary treatment. The molecular detection of COVID-19 infection in nasopharyngeal swab showed a negative result. MRI with contrast showed no abnormal brain structure. Her CSF analysis indicated a normal leucocyte count (360 cells/*μ*L), a high protein level (258.9 mg/dL), and a low level of glucose (23.4 mg/dL). While the result of the CSF culture revealed *S. enterica*, with pansensitive to almost all tested antimicrobials (Table 1). On the next day, the same organism (*S. enterica*) was isolated from the blood culture without any antimicrobial resistance. According to laboratory findings, she was diagnosed with Salmonella meningitis with the presence of *S. enterica* in the circulating blood (Salmonella bacteremia). Cefotaxime and amikacin were continued for 2 weeks after admission, at which time the CSF and blood culture were proved to be having no bacterial growth. After 2‐week antibiotic therapy, the female neonate was discharged without any sequelae.

### 3.2. The Genome Annotation and Genomic Features of the *S. enterica* Isolate

The *S*. *enterica* JZ01 genome was annotated utilizing the RAST tool kit (RASTtk) using genetic code 11, and the unique genome identifier of 28901.1487 was assigned. The genome of *S. enterica* JZ01 assembly yielded 38 contigs and a draft genome size of 4,542,804 bp, and the G + C content of the whole genome is 52.3%, which is similar to most *S. enterica* serotype Johannesburg strains. The chromosome consists of 4,501 predicted coding sequences (CDS), 3 rRNA genes, and 78 tRNA genes. WGS revealed that the isolate is *S. enterica* serotype Johannesburg, and it belongs to sequence type 515 (ST515) and core genome MLST (cgMLST 304742). The genome of our isolate harbored nine *Salmonella* pathogenicity islands (SPI-1, SPI-2, SPI-3, SPI-4, SPI-5, SPI-9, SPI-13, and SPI-14) and centisome 63 pathogenicity island (C63PI).

### 3.3. Antimicrobial Resistance (AMR) Genes and Virulence Factors Genes

**Figure 2:**
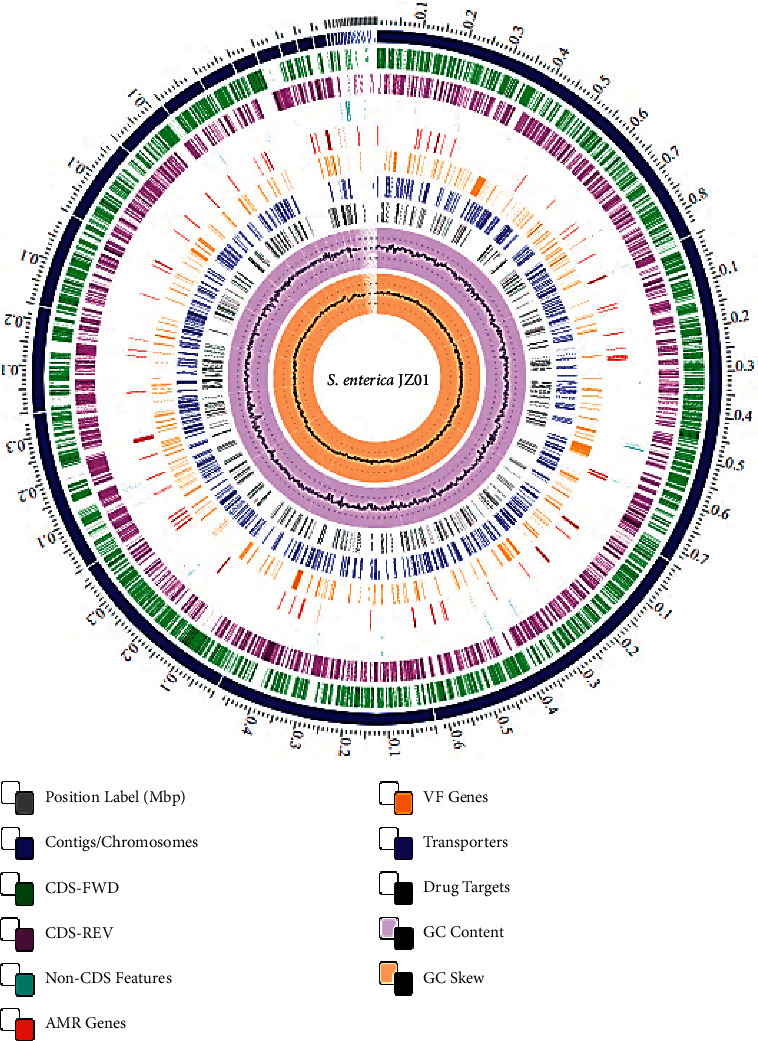
Circular graphical display of the distribution of the genome annotations of our *S. enterica* JZ01 strain. It includes, from outer to inner rings, the contigs (dark blue), CDS (coding DNA sequence) on the forward and reverse strand, RNA genes, CDS showing similarity to known antimicrobial resistance genes, CDS showing similarity to known virulence factors (yellow), GC content (pink), and GC skew (pale orange). The comprehensive genome analysis service of PATRIC (BV-BRC), which can be accessed at the website https://www.bv-brc.org/app/ComprehensiveGenomeAnalysis, on 6 October 2022.

PATRIC's genome annotation service utilizes a kmer-centric approach to identify various antimicrobial resistance gene variants. This service provides functional annotations, comprehensive antibiotic resistance mechanisms, drug classes, and occasionally, specific antibiotics associated with each AMR gene. It is noteworthy that the presence of AMR-related genes, even in complete form, indicates an antibiotic-resistant phenotype in the genome. It is crucial to consider specific AMR mechanisms, such as regulator modulating expression, efflux pump conferring, and targets in susceptible categories.  Table 2 provides a summary of annotated AMR genes and their corresponding resistance mechanisms in the genome. ResFinder also detected one acquired antimicrobial resistance gene aac(6′)-Iaa. Using a blast search, specific sources were used to identify the genes linked to pathogenicity and virulence. To expand the limited gene list in the database, various sources including CARD, NDARO, VFDB, and DrugBank were utilized. The analysis showed that the majority of the genes (681) were transporter genes, while the rest (117) were virulent factors. The major virulence genes that are associated with invasive infection include *ompA, fimH, orgA, yijP, fepA, pagC, AvrA, ppdD, iro (BN), inv (ABCEFGHIJ), sip (ABC), pho (PQ), mgt (BC), sse (BCDE), hil (ACDE), sop (ADE),* and *ssa (UTQP).* A circular graphical display of the distribution of the genome annotations is provided (Figure 2).

### 3.4. Comparison of the *S. enterica* JZ01 Genome with Other *S. enterica* subsp. Enterica Serovar Johannesburg Strains

**Figure 3:**
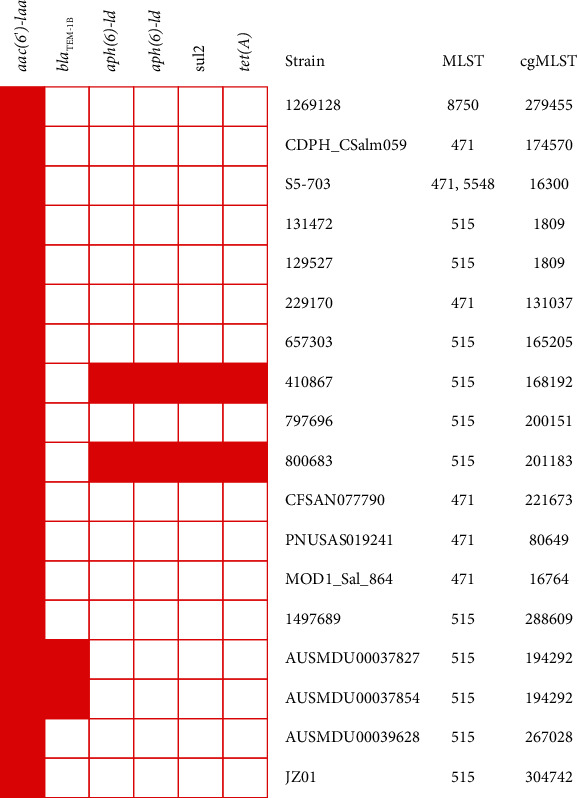
Genomic analysis of antimicrobial resistance genes (ARGs), multilocus typing (MLST), and core genome MLST (cgMLST) of *Salmonella enterica* subsp. enterica serovar Johannesburg isolated from Homo sapiens.

**Figure 4:**
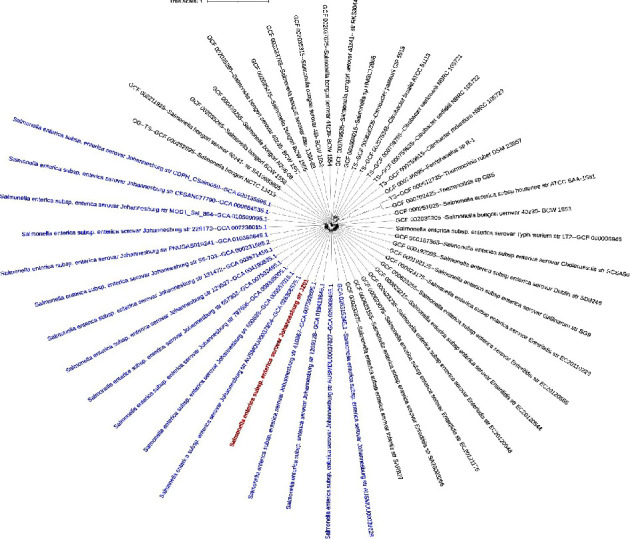
Maximum-likelihood phylogenetic analysis of the *Salmonella enterica* strain JZ01 (in red) based on multilocus sequence analysis from the genome sequences with *S. enterica* subsp. enterica serovar Johannesburg isolates (in blue) and other related species. The tree was constructed with the insert set of genomes into Species Tree v2.1.10 program with closely related genomes from the NCBI microbial genome database. The interactive Tree of Life tool was used to visualize the phylogenetic tree.

The NCBI bacterial genome database contained 1076 genomes (accessed 14 May 2023) of *Salmonella enterica* subsp. enterica serovar Johannesburg. Among them, 17 strains were isolated from Homo sapiens (human). ARGs analysis from the genome sequences of Johannesburg serotype revealed that most of the isolates were carrying only aac(6′)-Iaa gene causing aminoglycoside resistance consistent to *S. enterica* JZ01, where two strains were carrying *bla*_TEM−1B_ and the two other strains were carrying *aph*(6)-*Id*, *aph*(3″)-*Ib*, *sul*2, and *tet*(A) along with aac(6′)-Iaa gene (Figure 3). Moreover, the point mutation of *par*C p.T57S (ACC->AGC) causing quinolone resistance was commonly found in the analyzed genomes. The MLST analysis revealed that the majority of Johannesburg serovar isolates recovered from humans belonged to ST515 (11/18, 61.1%), followed by ST471 (4/18, 22.2%) (Figure 3). In the maximum-likelihood phylogenetic tree based on MLS analysis, *S. enterica* Johannesburg JZ01 unsurprisingly classified in one clade with *S. enterica* subsp. enterica serovar Johannesburg was isolated from Homo sapiens (Figure 4).

## 4. Discussion

The NTS species are known to cause asymptomatic infections (e.g., uncomplicated gastroenteritis) that rarely require antimicrobial treatment. However, invasive forms of infections such as bacteremia, meningitis, and osteomyelitis have been also reported [14, 15]. In the last decade, some cases of Salmonella meningitis in Saudi infants and children were documented [16], with only a rare incidence of NTS meningitis in female infant [17]. The emergence of another NTS strain causing neonatal meningitis in our region piqued our curiosity to conduct a whole genome analysis to discover the genomic characteristics of such an invasive strain. Livestock animals and their surrounding environment were considered the main reservoirs for *S. enterica* serotype Johannesburg [18, 19]. However, in this report, we isolated this serotype of salmonella from CSF and blood of neonate, which added to a few cases of human infection caused by Johannesburg serotype in the USA and Brazil, but these lacked clear clinical descriptions [20, 21]. Our *S. enterica* serotype Johannesburg JZ01 isolate belonged to clone type ST515, and this serotype was mainly linked to isolates from food and livestock animals in different countries [22–24]. Although the possibility of the current Salmonella infection being caused by the consummation of contaminated food is uncertain, it remains possible since our patient lives in a rural town in the Jazan region, which is famous for agriculture and livestock.

Although our isolate harboring *aac* (6′)*-Iaa* which confers resistance to aminoglycosides, the isolate was phenotypically sensitive to amikacin and tobramycin. A possible explanation for this might be that the aac (6′)-Iaa gene is chromosomally encoded in most *S. enterica*, and it is usually not expressed [25]. On the other hand, the importance of pathogenicity islands lies in their ability to harbor clusters of virulence genes that facilitate adhesion, invasion, and systemic dissemination. Most of these genes are located within highly conserved SPIs on the chromosome [26]. From 23 identified SPIs [27], nine SPIs (SPI-1–5, 9, 13–14, and C63PI) were detected in our isolate. Pathogenicity island (C63PI), an iron transport system in SPI-1, is an important part of the Salmonella chromosome which contributes to penetration into the host cell [28]. Furthermore, SPI-1 affects the whole process of Salmonella infection, including pathogen invasion, proliferation, and host responses, while SPI-2 confers the ability of *Salmonella* to survive in human macrophages [29]. Both SPI-1 and SPI- 2 encode different type III secretion systems (T3SS), which are responsible for delivering effector proteins into host cells and create proinflammatory responses [30]. Also, SPI-5 genes encoded the effector proteins for T3SS encoded by both SPI-1 and SPI-2 [31]. For bacterial invasiveness, genes encoded on SPI-3 and SPI-13 are pivotal for intracellular survival and intracellular viability, respectively [32, 33], while genes encoded on SPI-4 and SPI-9 are necessary for adhesion to epithelial cells [34, 35]. In addition, genes encoded on SPI-14 were found to play a role in the activation of SPI-1 genes and mediate bacterial invasion [36]. Penetrations of the blood-brain barrier (BBB) and cerebrospinal fluid barrier (BCSFB) represent the common invasion routes into the central nervous system (CNS) for bacterial pathogens [37]. The whole genome analysis of the current *S. enterica* JZ01 isolate revealed the presence of *fimH, ompA,* and *yijP*; these genes have been shown to play a major role in adhesion and invasion into barriers and cells of the central nervous system in *E. coli* [38–40]. Moreover, evading the innate immune system by resistance of the serum complement system is a major factor for the development of systemic infection by nontyphoidal Salmonella species [41]. The O-antigen of lipopolysaccharides (LPS) confers on the organism the capability to resist complement-mediated serum killing. In this study, we detected *rfe* and *rfaL*, which were found to be involved in the surface expression and function of O-antigen [42]. Moreover, the outer membrane protein *PagC* has been described that it confers a high level of resistance to the serum complements [43]. Furthermore, siderophores are important for bacterial growth in serum in the extracellular phase of salmonellosis. The major types of siderophores produced by Salmonella species include salmochelin, enterobactin, and aerobactin [44]. The existence of *fepA* and *iro (BN)* genes is required for the synthesis, secretion, and uptake of salmochelin. Moreover, enterobactin glycosylation, export, and utilization are activated by the presence of *iro(BN)* [45]. The *phoP* and *phoQ* genes are responsible for the control and regulate the expression of SPI-1 [46]. While *sip(ABC)* and *mgt(BC)* genes involve in the modification of lipid A which is related to Salmonella pathogenicity through intracellular survival and ion transport [26]. Furthermore, the role of the *orgA* gene was described among NTS for invasion and secretion systems [47]. The clusters of *inv*ABCEFGHIJ and *hil*ACD genes belong to SPI-1 and regulate the expression of T3SS1 (particularly *hil*A). These genes are essential for increasing the capability for invasion and colonization in host cells [48]. In addition, we identified *sop*ADE, effector genes of the T3SS1 that encode invasion-associated secreted effector proteins. Their activity role in increasing the virulence of *S. enterica* in humans was recently described [49]. The *avr*A gene, which is also present in SPI-1, was interfered in the host inflammatory response [50]. On the other hand, several clusters of virulence genes, which are located in SPI-2, were detected in our isolate. The *sscB, sseC, sseD, and sseE* genes encoding secreted effector proteins that are pivotal for *Salmonella* intracellular survival [51]. The *ssaU*, *ssaT*, *ssaQ,* and *ssaP* genes are known as components of T3SS2 and mediate the transport of bacterial virulence proteins into the host cell cytoplasm [30, 51]. Regarding immunocompetency of infants, defects in immunological mechanisms involving several cytokines and lymphocytes play an additional role in susceptibility to invasive NTS infection among infants [52]. In this study, other factors related to the immune system of our patient could influence her susceptibility to invasive infection. Therefore, the host immune determinants of NTS infection need further investigation.

In conclusion, we presented a genomic report of a rare case of NTS meningitis in an infant who is living in a rural town in Jazan region, Saudi Arabia. The infant developed meningitis as a complication of systemic infection probably due to her early age and an incomplete immune system. *S. enterica* serotype Johannesburg is a rare cause of human infection. Nontyphoidal Salmonella serotypes should be considered in newborns presenting with suspicious meningitis, especially those living in the rural areas and exposed to their environment. Several virulent genetic determinants within the genome of *S. enterica* serotype Johannesburg give this serovar the potential to cause invasive disease in humans. However, the capability of bacterial strains to cause systemic infection in infants is not sufficient suggesting that host factors and immune response play an additional role in developing the invasive infection. Considerably, more work needs to be done to define the role of host genetic determinants in iNTS infection. This will improve our understanding of the immunobiology of invasive Salmonella infection and will inform the delivery of novel control strategies.

## Figures and Tables

**Table 1:** Antibiotic susceptibility profile of *S. enterica* JZ01 isolate recovered from CSF and blood culture of the pediatric patient.

Antimicrobial agents	Cerebrospinal fluid		Blood	
	MIC (mg/L)	Interpretation	MIC (mg/L)	Interpretation
Amikacin	≤16	S	≤16	S
Amoxicillin/clavulanic acid	≤8/4	S	≤8/4	S
Ampicillin	≤8	S	≤8	S
Ampicillin/sulbactam	≤8/4	S	≤8/4	S
Cefepime	≤8	S	≤8	S
Cefotaxime	≤1	S	≤1	S
Ceftazidime	≤1	S	≤1	S
Ciprofloxacin	≤1	S	≤1	S
Ertapenem	≤0.5	S	≤0.5	S
Gentamicin	≤4	S	≤4	S
Imipenem	≤1	S	≤1	S
Meropenem	≤1	S	≤1	S
Piperacillin/tazobactam	≤16	S	≤16	S
Tigecycline	≤1	S	≤1	S
Tobramycin	≤4	S	≤4	S
Trimethoprim/sulfamethoxazole	≤2/38	S	≤2/38	S

**Table 2:** Genes associated with antimicrobial resistance mechanisms in the currently studied *S. enterica* JZ01 strain.

AMR mechanism	Genes
Antibiotic activation enzyme	KatG
Antibiotic inactivation enzyme,	AAC(6′)-Ic, f, g, h, j, k, l, r-z
Antibiotic resistance gene cluster, cassette, or operon	MarA, MarB, MarR
Antibiotic target in susceptible species	Alr, Ddl, dxr, EF-G, EF-Tu, folA, Dfr, folP, gyrA, gyrB, inhA, fabI, Iso-tRNA, kasA, MurA, rho, rpoB, rpoC, S10p, S12p
Antibiotic target protection protein	BcrC
Efflux pump conferring antibiotic resistance	AcrAB-TolC, AcrAD-TolC, AcrEF-TolC, AcrZ, EmrAB-TolC, MacA, MacB, MdfA/Cmr, MdtABC-TolC, MdtL, MdtM, MexPQ-OpmE,OprM/OprM family, SugE, TolC/OpmH
Gene conferring resistance via absence	gidB
Protein altering cell wall charge conferring antibiotic resistance	GdpD, PgsA
Regulator modulating expression of antibiotic resistance genes	AcrAB-TolC, EmrAB-TolC, H-NS, OxyR

## Data Availability

The data utilized to support the findings of this research can be obtained from the corresponding author upon request.
